# Role of Single-Photon Emission Computerised Tomography Versus Ultrasonography or 4D-Computed Tomography in the Management of Primary Hyperparathyroidism

**DOI:** 10.7759/cureus.29015

**Published:** 2022-09-10

**Authors:** Chirag Pereira

**Affiliations:** 1 General Surgery, Royal Lancaster Infirmary, Lancaster, GBR

**Keywords:** ultra-sonography, 4d-ct, single-photon emission computed tomography, parathyroid gland adenoma, primary hyperparathyroidism

## Abstract

Introduction

The aim of the current study was to determine the diagnostic accuracy of three commonly used localization modalities for parathyroid adenomas, i.e., single-photon emission computerised tomography/computed tomography (SPECT/CT), ultrasound (USG) and 4D-computed tomography (4D-CT), especially when used in combination.

Methods

Medical records of patients diagnosed with primary hyperparathyroidism were reviewed from January 2015 to December 2020. Intra-operative findings were compared with preoperative localization studies (USG, SPECT/CT and 4D-CT) in order to determine sensitivity, specificity and accuracy of these studies.

Results

One hundred eighty-nine medical records were reviewed. SPECT/CT has a sensitivity of 60.51% and a diagnostic accuracy of 60.21%. USG had the lowest sensitivity of 49.36% with a diagnostic accuracy of 51.6%. 4D-CT had the highest sensitivity of 82.72%, a specificity of 56% and a diagnostic accuracy of 76.42%. When SPECT/CT was used in combination with USG the sensitivity was significantly higher (p=0.0001) at 69.54% and when SPECT/CT was used in combination with 4D-CT the sensitivity was significantly higher at 91.4% (p=0.0001).

Conclusions

SPECT/CT was more sensitive and accurate as compared to USG but when they were used together the sensitivity was significantly higher. Superior preoperative localization was provided by 4D-CT as compared to SPECT/CT.

## Introduction

One of the most common endocrine diseases is primary hyperparathyroidism (PHPT), which results from excessive production of parathyroid hormone (PTH). The most common etiology causing PHPT is parathyroid adenoma followed by hyperplasia and the least common cause is parathyroid cancer [1]. Diagnosis of PHPT is based on biochemical blood investigations showing elevated levels of PTH and serum calcium when other causes of hypercalcemia have been ruled out. PHPT presents clinically with a manifestation of symptoms, which are often described as “stones, bones, abdominal groans, thrones and psychic overtones” [2]. Operative management of PHPT is dependent on successful excision of the hyperactive parathyroid gland. The standard approach to the management of parathyroid adenoma was bilateral neck exploration but nowadays a minimally invasive approach is commonly being performed. The success of the minimally invasive approach is dependent on the preoperative localization of the abnormal parathyroid gland.

Numerous modalities of imaging have been utilized when it comes to parathyroid localization. The least invasive among these is ultrasound (USG) neck and successful identification of the parathyroid gland is dependent on the operator's experience, size of the adenoma and co-existing thyroid lesions [3,4]. One of the most commonly used localization studies for the parathyroid disease is sestamibi scintigraphy. This test makes use of a radioisotope that gets accumulated in cells having a high metabolic rate such as parathyroid adenoma. The most commonly used radioisotope agent is technetium-99m methoxy isobutyl isonitrile (99mTc-MIBI) [5]. Very often single photon emission computed tomography (SPECT) is combined with computed tomography (CT) which aids in anatomical localization [6,7]. The limitation of SPECT/CT is difficulty in localization when there is multi-gland disease, small adenomas and concurrent thyroid nodules present [8].

The protocol in a few centres where USG and SPECT/CT are discordant is to use 4D-CT to aid in localisation. Conventional CT is not preferred for localization as it is difficult to differentiate lymph nodes from parathyroid adenomas. 4D-CT on the other hand involves taking images during a late contrast washout phase. Parathyroids appear to be highly vascular structures with variable contrast enhancement and early wash-out of contrast on 4D-CT [9].

The aim of the current study was to determine the diagnostic accuracy of three commonly used localization modalities for parathyroid adenomas, i.e., SPECT/CT, US and 4D-CT, especially when used in combination.

## Materials and methods

Medical records of patients diagnosed with PHPT were reviewed from January 2015 to December 2020 at Royal Lancaster Infirmary. Histology (frozen section and formal histology) and radiological data (USG, SPECT/CT and 4D-CT) were obtained from an online database. Operative notes were reviewed in order to determine the location of an abnormal parathyroid gland. The position of abnormal parathyroid glands was classified as either left upper, left lower, right upper, right lower or ectopic in order to assess the concordance between intra-operative findings and preoperative imaging. Cases of double adenoma were considered to be two separate results for analysis. Parathyroid adenoma, hyperplasia and carcinoma were considered as pathological parathyroid tissue. For patients requiring re-exploration, only data from initial surgery was collected. Patients that did not have clear operative notes, missing radiological reports or indeterminate histology were excluded from the study. Since this was a retrospective study informed consent was not required and the study was ethically approved and granted by the regional governance committee.

Surgical technique

Surgery was performed by two surgeons who are experts in their field. A minimally invasive approach with a 3-4cm midline collar incision and focused approach on the side was performed based on preoperative localization. Intra-operative PTH monitoring as well as a frozen section was performed in order to confirm successful excision. Intra-operative PTH levels were checked 10 minutes following the successful removal of a suspected parathyroid gland. If the value of PTH dropped by > 50% of the preoperative level, it was considered to be a successful excision. When the frozen section was unavailable the excised gland was sent for formal histology. If the gland was not located on a minimally invasive approach, a standard neck exploration was carried out which involved extension of neck incision and exploration of all neck quadrants for suspected parathyroid adenoma.

Statistical analysis

Categorical data were expressed as percentage and number. Sensitivity, specificity, positive predictive value, negative predictive value and diagnostic accuracy were calculated for USG, SPECT/CT and 4D-CT. Combined sensitivity and specificity were determined for USG and SPECT/CT and for SPECT/CT and 4D-CT groups. McNemar’s test was used to determine significance between paired nominal data and P-values < 0.05 were considered statistically significant.

## Results

Two hundred and twelve medical records were reviewed from January 2015 to December 2020. Of these, 23 records were excluded due to indeterminate cytology and unclear reports on localization. The age of patients ranged from 34 to 89 years with larger number of females (80.4%) as compared to males. There were 163 (86.2%) cases of single adenomas, four (2.1%) cases of parathyroid hyperplasia and two (1.1%) cases of double adenoma. There was one ectopic adenoma found in the superior mediastinum. Normal parathyroid histology and no pathological tissue were seen in 20 (10.6%) cases (Table 1).

**Table 1:** Patient demographics

Characteristics:	
Mean Age (Range)	63.9 (34-89)
Male (%)	37 (19.6%)
Female (%)	152 (80.4%)
Histological diagnosis	
Single adenoma	163
Double adenoma	2
Hyperplasia	4
Normal histology	4
No pathological tissue	16
Preoperative imaging	
Number of USG	185 (98.8%)
Number of SPECT/CT	189 (100%)
Number of 4D-CT	105 (55.7%)

All 189 patients included in this study underwent SPECT/CT. Of these, 185 patients underwent ultrasound of the neck, and 105 patients underwent 4D-CT for localization. When looking at localization studies independently, SPECT/CT has a sensitivity of 60.51% a specificity of 58.8% and a diagnostic accuracy of 60.21%. USG had the lowest sensitivity of 49.36% with a diagnostic accuracy of 51.6%. 4D-CT had the highest sensitivity of 82.72%, a specificity of 56% and a diagnostic accuracy of 76.42% (Table 2).

**Table 2:** Individual localization studies: sensitivity, specificity and diagnostic accuracy *n refers to a number of adenomas scanned, not the number of patients PPV: positive predictive value, NPV: negative predictive value

	SPECT/CT (*n =191)	USG (*n =186)	4D-CT (*n = 107)
Sensitivity	60.51%	49.36%	82.72%
Specificity	58.82%	62.50%	56%
PPV	87.16%	86.52%	85.9%
NPV	24.39%	20.2%	50%
Accuracy	60.21%	51.6%	76.42%

Paired sensitivity of USG and SPECT/CT was also determined as shown in Table 3. Of the 186 lesions scanned by both USG and SPECT/CT, SPECT/CT had a higher sensitivity as compared to USG (59.48% vs 50%) but this was not statistically significant. (p=0.05, χ2=3.841). When SPECT/CT was used in combination with USG the sensitivity was significantly higher (p=0.0001, χ2=10.56) at 69.54% as compared to SPECT/CT used individually. The combination also had a higher diagnostic accuracy of 67.2% (p=0.0002, χ2=13.4%) (Table 3).

**Table 3:** Combined SPECT/CT and USG: sensitivity, specificity and diagnostic accuracy *n refers to the number of adenomas scanned, not the number of patients

Paired SPECT/CT and USG for Parathyroid Lesions (*n= 186)					
	SPECT/CT	USG	P-value	SPECT/CT + USG	P-value
Sensitivity	59.48%	50%	0.05	69.54%	0.001
Specificity	51.52%	58.82%	0.24	57.14%	0.24
Accuracy	58.06%	51.6%	0.1447	67.2%	0.0002

Of the 107 lesions scanned by both SPECT/CT and 4D-CT, 4D-CT had a significantly higher sensitivity as compared to SPECT/CT alone (83.75% vs 37.5%). When SPECT/CT was used in combination with 4D-CT the sensitivity was significantly higher at 91.4% (p=0.0001, χ2= 37.2) and had a diagnostic accuracy of 83.96% as compared to SPECT/CT alone which had an accuracy of 41.5% (p=0.0001, χ2=40.19) (Table 4).

**Table 4:** Combined SPECT/CT and 4D-CT: sensitivity, specificity and diagnostic accuracy *n refers to the number of adenomas scanned, not the number of patients

Paired SPECT/CT and 4D-CT for Parathyroid Lesions (*n= 107)					
	SPECT/CT	4D-CT	P-value	SPECT/CT + 4D-CT	P-value
Sensitivity	37.5%	83.75%	0.0001	91.46%	0.0001
Specificity	61.11%	50%	0.61	58.33%	0.24
Accuracy	41.5%	75.47%	0.0001	83.96%	0.0001

## Discussion

Preoperative localisation of abnormal parathyroid glands is an essential component in planning and successfully carrying out a minimally invasive approach to parathyroidectomy. A minimally invasive approach is preferred to the standard neck exploration as surgeons have reported shorter hospital stays, lower rates of post-operative hypocalcemia, better cosmesis and less post-operative pain [10]. There is no single best localisation study, but surgeons rely on a combination of these studies. Most surgeons advocate the use of two localization investigations to detect the position of adenomas and almost all advocate the use of intraoperative PTH monitoring [11]. National Institute of Health and Care Excellence (NICE) recommends USG as an initial investigation for localization and a second modality (such as a sestamibi scan) if it aids in guiding the surgical approach [12].

Studies have shown that the addition of USG to scintigraphy improves the sensitivity and accuracy of the test [13,14]. This is also true in the current study where the combined localization tests had higher sensitivity and diagnostic accuracy as compared to individual tests. The addition of USG to sestamibi resulted in the conversion of 13 out of 62 false negatives into true positives. The sensitivity of USG in the current study was 49.36% which is lower compared to other studies, which showed a sensitivity that ranged from 55% to 83% [15]. USG neck is valuable in assessing the thyroid gland in the same setting for nodules but is of limited value when it comes to visualizing ectopic glands in the mediastinum.

In the current study, 4D-CT had a significantly higher sensitivity as compared to SPECT/CT (83.75% vs 37.5%). Rodgers et al. [16], in their study, evaluated 75 patients for parathyroid localization with 4D-CT and sestamibi. They found CT to have a sensitivity of 70% as compared to sestamibi which had a sensitivity of 33%. This is comparable to the present study. Yeh et al. [17] in their study evaluated a much larger population and found CT to have a higher sensitivity than sestamibi (79% vs 58%, respectively). Furthermore, in the current study, CT had a higher diagnostic accuracy as compared to SPECT. In a study by Kedarisetty et al. [13], CT had a higher diagnostic accuracy as compared to SPECT but failed to show a significant association (p=0.68).

The main limitation of the current study is that it is retrospective, hence certain patients that had localization studies performed did not subsequently undergo surgery and were excluded from the analysis. There is a degree of bias in reporting of results as SPECT/CT may have been made available to the radiologist at the time of evaluating 4D-CT.

## Conclusions

The current study evaluates and compares the sensitivity, specificity and diagnostic accuracy of SPECT/CT, USG and 4D-CT individually and in combination. SPECT/CT was more sensitive and accurate as compared to USG but when they were used together the sensitivity was significantly higher. Superior preoperative localization was provided by 4D-CT as compared to SPECT/CT. 4D-CT should be considered in those patients having discordant USG and SPECT/CT in localizing parathyroid adenomas.
